# Mixed Classes in Medical Education

**Published:** 1916-07-08

**Authors:** 


### MIXED CLASSES IN MEDICAL EDUCATION.

The pressure of the war on the medical profession has, among other consequences, led to a considerable increase in the number of women medical students, and this in turn has necessitated a consideration of the facilities which exist for the professional education of these students. Hitherto, at least for the most part, separate accommodation has been provided by special schools and selected hospitals, and on the whole this arrangement has worked satisfactorily. The disadvantage of the plan is that it restricts the choice of the women to a limited number of teachers, and hence their educational opportunities are, generally speaking, less broad than those enjoyed by men. Separation has doubtless much to be said in its favour, but the handicap just mentioned exists and has a practical as well as a sentimental application. With enlarging numbers there is little wonder that in some quarters has arisen a demand for a larger liberty and for a full equality of choice in the selection of an area of education. Again, with the rush of new entries some of the women's colleges find their classes and their laboratories overcrowded and are compelled to look outside for the needed accommodation. This strain is likely to become increasingly severe as the new students begin to need clinical instruction, for while added numbers in a classroom can perhaps be accommodated, teaching in hospital wards, if it is to be of practical value, must necessarily be confined to a small number. Clinical training, in the very nature of things, means the opportunity for the student personally to observe the facts of disease as these are displayed by individual patients, and therefore the size of a clinical class must be always a limited one.

In these circumstances some of the medical schools hitherto confined to the education of men have been urged to open their doors to women, and the request has in certain instances met with a favourable response. This, of course, means the establishment of mixed classes. To such classes objections have been urged, and not the least emphatic of these have come from persons specially interested in the medical education of women. These objections are now being subjected to the test of experiment, and the outcome will be watched with interest. Perhaps the general expectation is that if failure results it will be due to the attitude of the women students. But there is at least the possibility that hostility may come from the other side. Not that the men will be wanting in generosity, but some of them will feel that the introduction of women destroys the equality which ought to reign among students. A man among his fellows is free to seize what chances come within his reach even though the process means some elbowing out of his fellows. But not unreasonably he may shrink from treating a woman in so cavalier a fashion. Hence he is reduced to the alternative either of missing a chance which might have been to his advantage or of acting below his usual standard of chivalry. There will certainly be some who will object to have such a choice forced upon them, and who will therefore prefer a school where they may escape it. It follows that even though mixed classes find favour with the women students they may still come to grief before the not ungenerous objections of the men. Perfect equality is hard to compass, and Nature, though expelled for a season, has a habit of returning to vindicate its claim.

